# Prognostic value and their clinical implication of 89-gene signature in glioma

**DOI:** 10.18632/oncotarget.9983

**Published:** 2016-06-13

**Authors:** Muhammad Shahid, Kyoung Min Cho, Minh Nam Nguyen, Tae Gyu Choi, Yong Hwa Jo, Saurav Nath Aryal, Ji Youn Yoo, Hyeong Rok Yun, Jae Woong Lee, Young Gyu Eun, Ju-Seog Lee, Insug Kang, Joohun Ha, Hwi-Joong Yoon, Si-Young Kim, Sung Soo Kim

**Affiliations:** ^1^ Department of Biomedical Science, Graduate School, Kyung Hee University, Seoul, Republic of Korea; ^2^ Department of Internal Medicine, Graduate School, Kyung Hee University, Seoul, Republic of Korea; ^3^ Department of Biochemistry and Molecular Biology, School of Medicine, Kyung Hee University, Seoul, Republic of Korea; ^4^ Department of Otolaryngology-Head and Neck Surgery, Kyung Hee University Medical Center, Seoul, Republic of Korea; ^5^ Department of Systems Biology, Division of Cancer Medicine, The University of Texas MD Anderson Cancer Center, Houston, Texas, USA

**Keywords:** glioma, gene expression profile, prognosis, chemosensitivity

## Abstract

Gliomas are the most common and aggressive primary tumors in adults. The current approaches, such as histological classification and molecular genetics, have limitation in prediction of individual therapeutic outcomes due to heterogeneity within the tumor groups. Recent studies have proposed several gene signatures to predict glioma's prognosis. However, most of the gene expression profiling studies have been performed on relatively small number of patients and combined probes from diverse microarray chips. Here, we identified prognostic 89 common genes from diverse microarray chips. The 89-gene signature classified patients into good and bad prognostic groups which differed in the overall survival significantly, reflecting the biological characteristics and heterogeneity. The robustness and accuracy of the gene signature as an independent prognostic factor was validated in three microarray and one RNA-seq data sets independently. By incorporating into histological classification and molecular marker, the 89-gene signature could further stratify patients with 1p/19q co-deletion and IDH1 mutation. Additionally, subset analyses suggested that the 89-gene signature could predict patients who would benefit from adjuvant chemotherapy. Conclusively, we propose that the 89-gene signature would have an independent and accurate prognostic value for clinical use. This study also offers opportunities for novel targeted treatment of individual patients.

## INTRODUCTION

Gliomas are the most common and aggressive primary tumors in adults [1, 2]. Based on the histological appearance, gliomas can be subdivided into astrocytomas, oligodendrogliomas, and mixed oligoastrocytomas [3]. In 2007, World Health Organization (WHO) further subclassified gliomas into grade I (pilocytic astrocytomas), grade II (diffuse infiltrating low-grade gliomas), grade III (anaplastic gliomas, AA), and grade IV (glioblastomas multiforme, GBM) depending on the degree of aggressiveness [4].

In current clinical practice, histological classification is a critical prognostic factor that determines the choice of therapy. The response to therapy and the overall survival (OS) of glioma patients varies in different histological subtypes and grades [4]. Generally, oligodendrogliomas have a better prognosis than mixed oligoastrocytomas, and astrocytomas have the worst prognosis [3]. The median survival time is only 1.6 and 0.4 years for grades III and IV gliomas, respectively [5]. However, histological classification has a limited role in the treatment decision and prediction of individual outcomes due to their subjective criteria and lack of reproducibility [6]. Therefore, recent molecular genetic analyses, such as 1p/19q co-deletion and isocitrate dehydrogenase 1 (IDH1) mutation, have been extensively investigated to develop more objective approaches. Unfortunately, molecular genetic approaches are also limited by the heterogeneity within the tumor groups [7].

Recently, microarray gene analytic tools have been developed for diverse cancers for diagnosis, prognosis or prediction of therapeutic response [8–12]. In various cancers, there are several reports on gene expression profiles for its classification, prognosis and identification of biological processes including cell differentiation and proliferation [13–16]. However, most of the studies on glioma were performed on small number of patients and combined probes of diverse microarray chips. If the patients number is small, gene-expression profiles can vary according to the microarray platform and the analytic strategy, resulting in increased bias [17]. In previously published reports, the gene expression analyses on glioma also have a limitation due to the lack of reflected histology and molecular heterogeneity. In addition, well-defined target genes which predict chemotherapy response in glioma are rare.

In this study, we identified 89 common genes from diverse microarray chips using relatively large number of patients and investigated whether the 89-gene signature could be robustly validated in independent and combined data sets. Moreover, we attempted to establish a prediction model by incorporating the 89 gene set into other clinicohistological factors and molecular markers. Herein, we reported the 89-gene signature that could predict the survival of patients as well as their response to chemotherapy.

## RESULTS

### Significant association of prognosis with two groups found by hierarchical clustering

We selected four microarray and one RNA-seq data sets, which consisted of GSE16011, TCGA, GSE4412 and GSE4271. GSE16011 was used as the training data set because it had enough number of patients with clinical information such as grade, chemotherapy, radiotherapy, and gene mutation. Detailed informations for these data sets were described in Materials and Methods (Table 1).

**Table 1:** Clinical and histological characteristics of patients with glioma

Variable	EUMC GSE16011	TCGA	UCLA GSE4412	MDAS GSE4271
Patients (n)	264	342	85	77
Male	177	210	32	52
Female	87	132	53	25
Age (years)	51 (11–82)	59 (10–89)	42 (18–82)	45 (22–82)
Grade (n)				
I	6			
II	23			
III	84		26	21
IV	151	342	59	56
Adjuvant chemotherapy (n)				
Yes	27	258		
No	168	61		
N/A	69	23		
Radiotherapy				
Yes	193	280		
No		51		
N/A	71	11		

**Figure 1:**
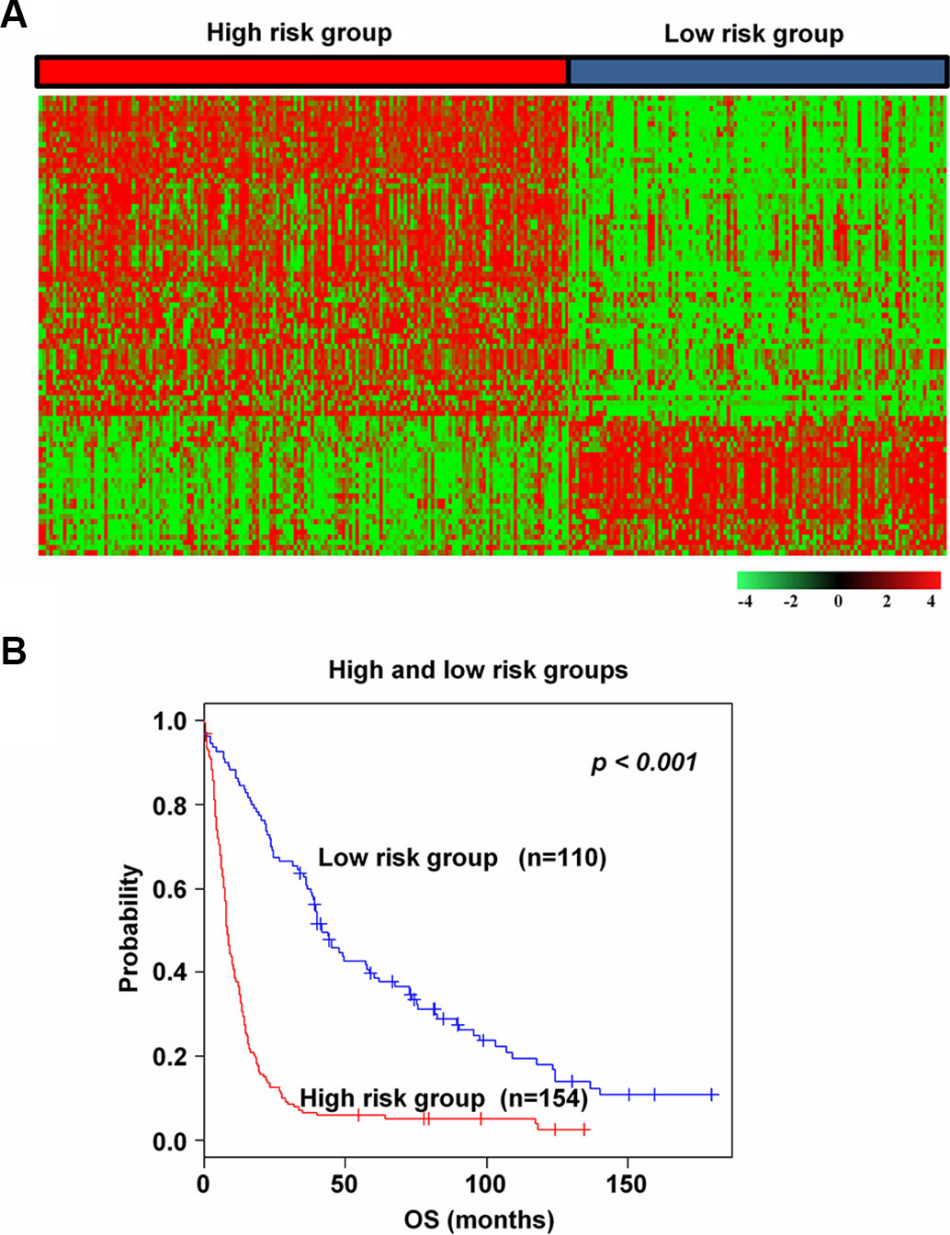
Survival analysis of the training data set (**A**) The heatmap of the median centered 89 genes' expression profiles (red, relative high expression; green, relative low expression) between high and low risk groups in the training data set. (**B**) Kaplan-Meier plots of overall survival (OS) of the two groups in the training data set. The *p* values were computed by the log-rank test.

To generate a potential molecular classifier of glioma, genes with an expression level of at least 2-fold difference relative to median value were selected from the training data set. Then, hierarchical clustering was performed and the results revealed two major groups (*n* = 154 and *n* = 110) of glioma that differed in gene expression patterns (Supplementary Figure S1). Next, a stringent threshold cut-off (*p* < 0.001 and 2.5 fold difference) was applied, and 129 genes whose expression was tightly associated with the two groups were selected (Supplementary Figure S2). Because 89 genes were common among all training and validation data sets, they were used as prognostic signature (Figure 1A). To evaluate groups' prognosis, Kaplan–Meier survival curves were plotted and the log-rank test showed significant difference in overall survival (OS) (*p* < 0.001, Figure 1B). Patients were classified into high and low risk groups by risk relied on differences in OS in the training set.

A previous report showed that patients with grade II survived for more than 5 years, whereas the median survival time of patients in grade III was 1.6 years, indicating malignant gliomas [5]. The Kaplan–Meier plots and the log-rank test showed significantly different OS in all of the grades in training data set (*p* < 0.001, Supplementary Figure S3). Unfortunately, patients in grades II and III could not be stratified in the training set contrary to general findings in histological classification. However, our gene signature demonstrated a prognosticvalue beyond the standard clinical classification of grades.

### Prognostic gene signature and clinical relevance

To investigate the association between prognostic gene signature and clinicohistological characteristics, including gender, grade and survival, Chi-square (*χ*^2^) test was performed in training data set (Table 2). The grade (*p* = 0.03) was significantly correlated to our gene signature, while other covariates were not associated. To evaluate prognostic accuracy of the 89-gene signature in combination with covariates, including patient age at diagnosis, gender, grade, and adjuvant chemotherapy, univariate and multivariate Cox proportional hazards regression analyses were performed using the training data set. In both of univariate and multivariate analyses, grade was significantly associated with OS (HR: 2.66, 95% CI 2.11–3.36, *p* = 1.2e–16 and HR: 1.65, 95% CI 1.24–2.21, *p* = 0.001, respectively). Notably, the 89-gene signature showed stronger prognostic ability over histological grade (HR: 0.27, 95% CI 0.20–0.36, *p* = 3.8e–18 and HR: 0.23, 95% CI 0.15–0.34, *p* = 8.8e–18, respectively) in both of univariate and multivariate analyses (Table 3). No significant difference was obtained in other covariates.

**Table 2:** Clinical and histological feature of two subgroups of gliomas patients in the EUMC (*n* = 264)

Variable	High risk group	Low risk group	*p*-value
No. of Patients	154	110	0.42
Male	102	75	
Female	52	35	
Grade			0.03
I	5	1	
II	22	1	
III	66	18	
IV	61	90	

**Table 3:** Univariate and multivariate Cox proportional hazard regression analyses of OS in the EUMC (*n* = 264)

Variable	Univariate		Multivariate	
	HR (95% CI)	*p* Value	HR (95% Cl)	*p* Value
Gender (Male or Female)	0.91 (0.69–1.19)	0.511	1.02 (0.74–1.40)	0.88
Age (< 40, > 40)	2.77 (2.02–3.78)	< 0.001	1.75 (1.22–2.51)	0.002
Adjuvant chemotherapy	1.50 (0.96–2.32)	0.070	1.50 (0.96–2.35)	0.070
Grade (I, II, III, IV)	2.66 (2.11–3.36)	1.2e–16	1.65 (1.24–2.21)	0.001
Gene signature (High/Low risk group)	0.27 (0.20–0.36)	3.8e–18	0.231 (.15–.34)	8.8e–18

### Validation of prognostic gene expression signature in independent validation data sets

**Figure 2:**
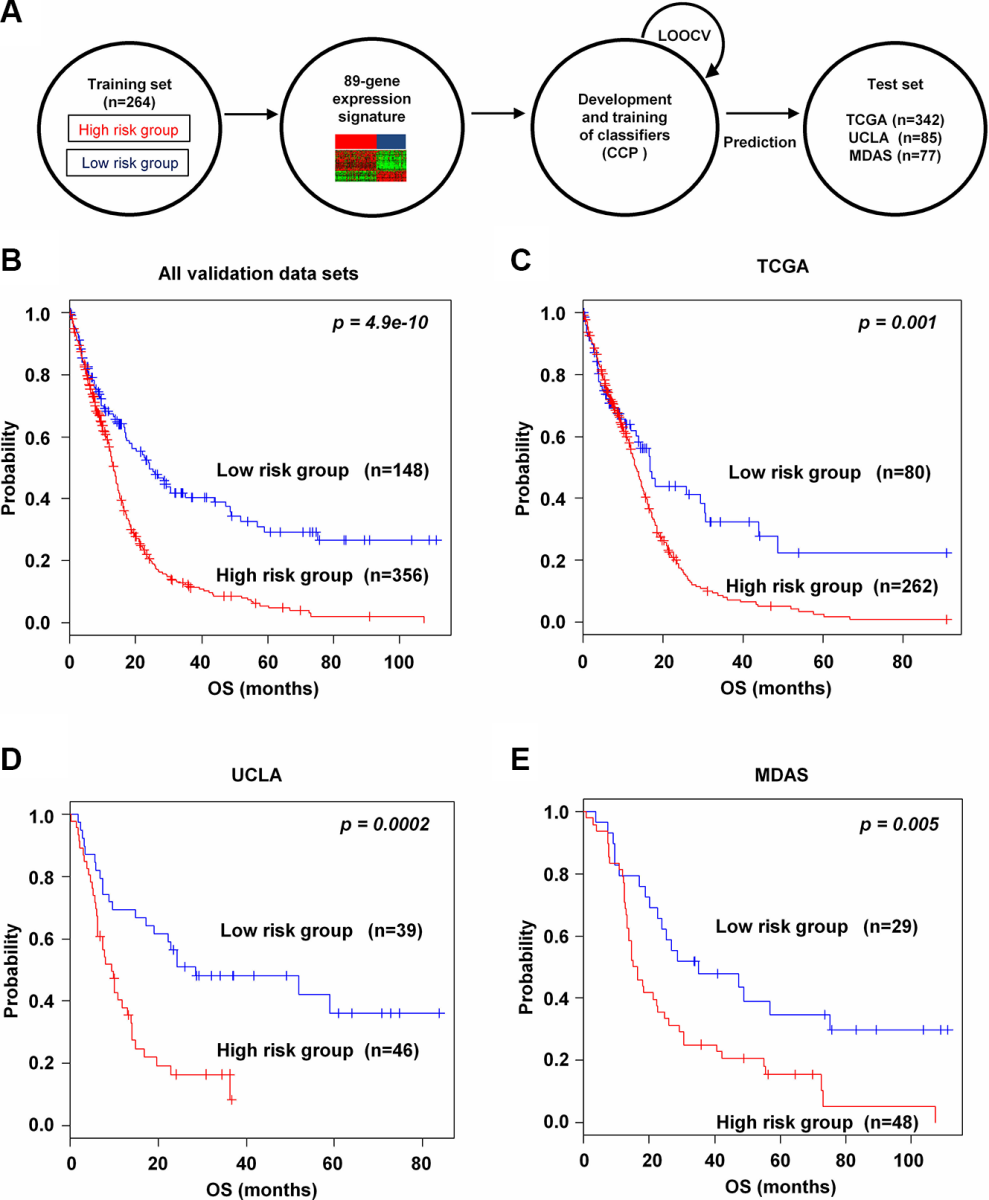
Prognostic significance of the 89-gene signature in independent validation data sets (**A**) Schematic overview of the strategy used for the construction of the prediction model and evaluation of predicted outcomes in three independent data sets by the 89-gene signature. (**B**) All combined validation data sets were stratified by the 89-gene signature into two groups. The *p* values were computed by the log-rank test. (**C**–**E**) Kaplan-Meier survival plots of overall survival (OS) of the two groups in three independent data sets: TCGA, UCLA, and MDAS.

To evaluate the robustness of the newly identified 89-gene signature, validation processes were done on three independent microarray and one RNA-seq data sets of glioma. A flow chart of the validation procedure was described in Figure 2A. During leave-one-out cross-validation (LOOCV), the specificity and the sensitivity for predicting groups in all validation data sets were 0.94 and 0.93, respectively. To identify whether the gene signature could be a more accurate prediction model, we validated in the combined three validation data sets. As expected, the gene signature significantly classified patients into high and low risk groups (*p* = 4.9e–10, Figure 2B). Also, Kaplan-Meier plots predicted significant differences in prognosis in all independent validation data sets: TCGA (*p* = 0.001, Figure 2C), UCLA (*p* = 0.0002, Figure 2D) and MDAS (*p* = 0.005, Figure 2E). We also validated RNA-seq data from TCGA based on the 89-gene signature (*p* = 0.035, Supplementary Figure S4).

### Association of the 89-gene signature with molecular pathway and mutation

**Figure 3:**
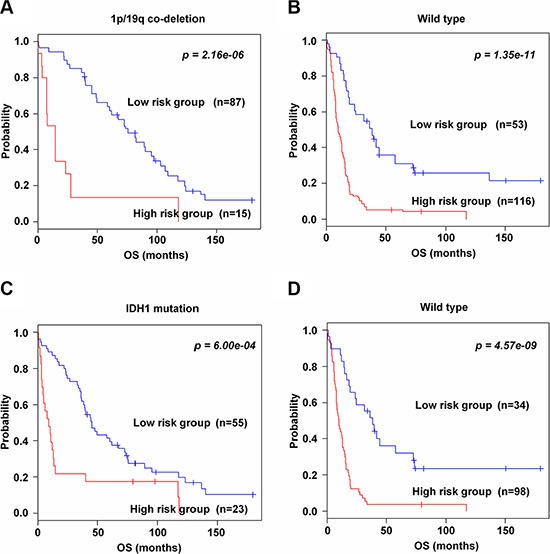
Significant association of the 89-gene signature with molecular pathways and mutation in the training data set (**A–B**) Kaplan-Meier curves of patients in 1p/19q co-deletion and wild type groups. (**C–D**) IDH1 mutation and wild type groups in the training data set. Patients were classified by the 89-gene signature. The *p* values were computed by the log-rank test.

To investigate whether the 89-gene signature could further stratify glioma patients associated with 1p/19q and IDH1 status, subset analyses were performed only in the training data set, because of the available clinical information. In both 1p/19q co-deletion and wild type groups, the 89-gene signature successfully classified patients into high and low risk groups (*p* = 2.16e–06 and *p* = 1.35e–11, Figure 3A–3B, respectively). Similarly, the 89-gene signature significantly classified patients with IDH1 mutation (*p* = 6.00e–04, Figure 3C) and wild type groups into high and low risk groups (*p* = 4.57e–09, Figure 3D). Consistent with previous reports demonstrating that the 1p/19q co-deletion and IDH1 mutation generally have favorable prognosis [18–20], our study classified most patients in these groups into low risk, eighty seven (85.3%) and fifty five (70.5%) patients, respectively. On the contrary, the 1p/19q and IDH1 wild type groups were classified into high risk, one hundred sixteen (68.6%) and ninety eight (74.2%) patients, respectively.

### Subset classification of age groups by the 89-gene signature

**Figure 4:**
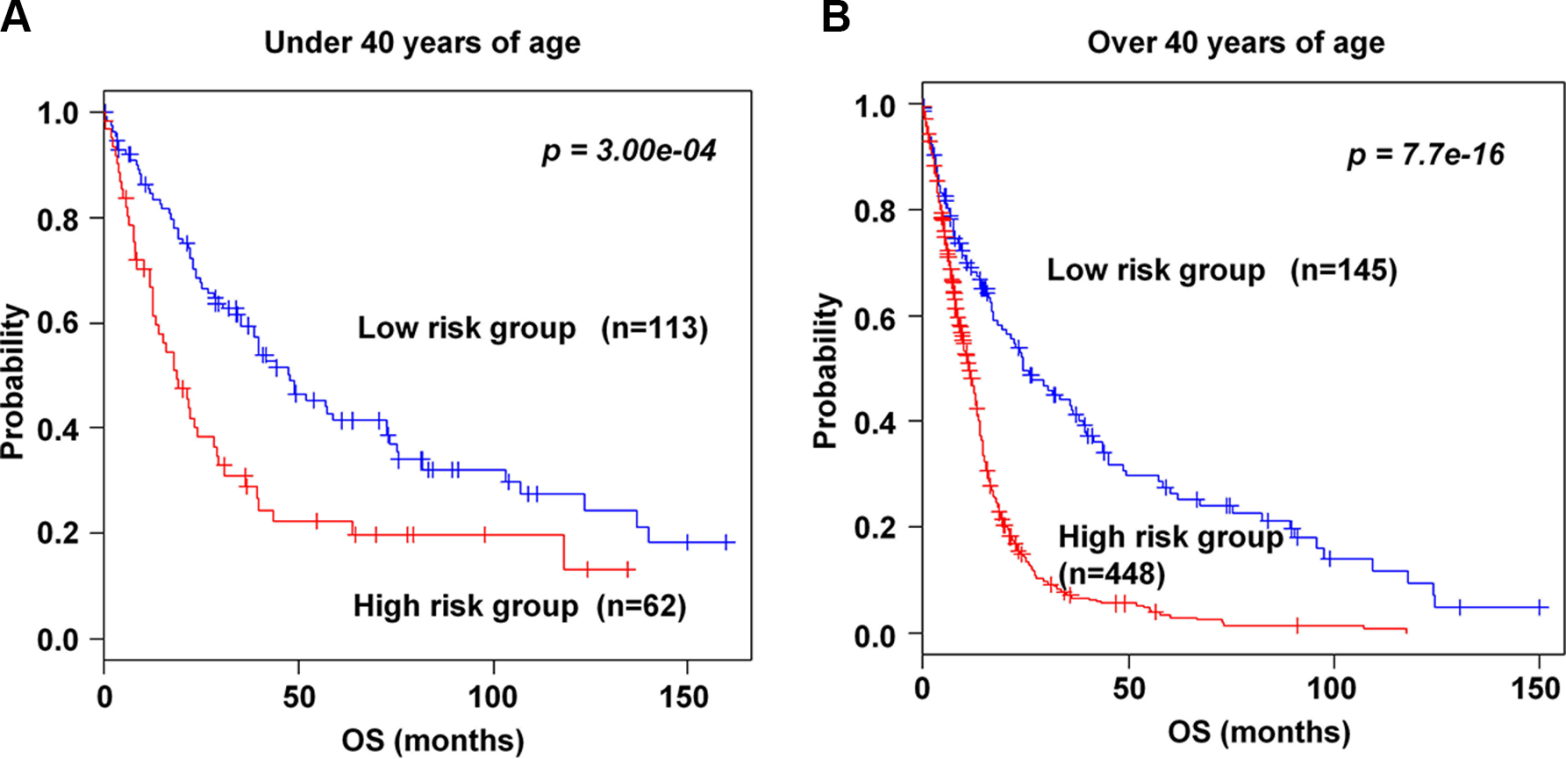
Kaplan-Meier survival analysis of the 89-gene signature in age (**A**) Patients under 40 years of age group in the combined training and validation data sets were stratified into high and low risk groups. (**B**) Patients over 40 years of age group in the combined training and validation data sets were stratified into high and low risk groups. The *p* values were computed by the log-rank test.

To investigate the association of the 89-gene signature with age, patients were classified into under 40 (young patients) and over 40 years of age (old patients) groups. Patients who are diagnosed with gliomas, younger age at diagnosis is a strong predictor of longer patient survival. In both age groups, the 89-gene signature significantly stratified patients in the combined training and validation data sets into high and low risk groups (*p* = 3.00e–04 and *p* = 7.7e–16, Figure 4A–4B, respectively). Consistent with recent report showing that patients under 40 years old have more favorable prognosis than patients over 40 years old [21], our study classified most patients under 40 years as low risk and over 40 years of age as high risk. One hundred thirteen (64.6%) patients were classified into low risk in under 40 years of age and 448 (75.5%) patients were classified into high risk in over 40 years of age group. In addition, the gene signature significantly classified patients in over 40 years, even not in under 40 years, in the training set (*p* < 0.001, Supplementary Figure S5A–S5B). Similar results were shown in patients in both groups in validation data sets (*p* = 6.00e–04 and *p* = 0.006, Supplementary Figure S5C-S5D, respectively).

### Prognostic subclassification of patients with grades III and IV by the 89-gene signature

**Figure 5:**
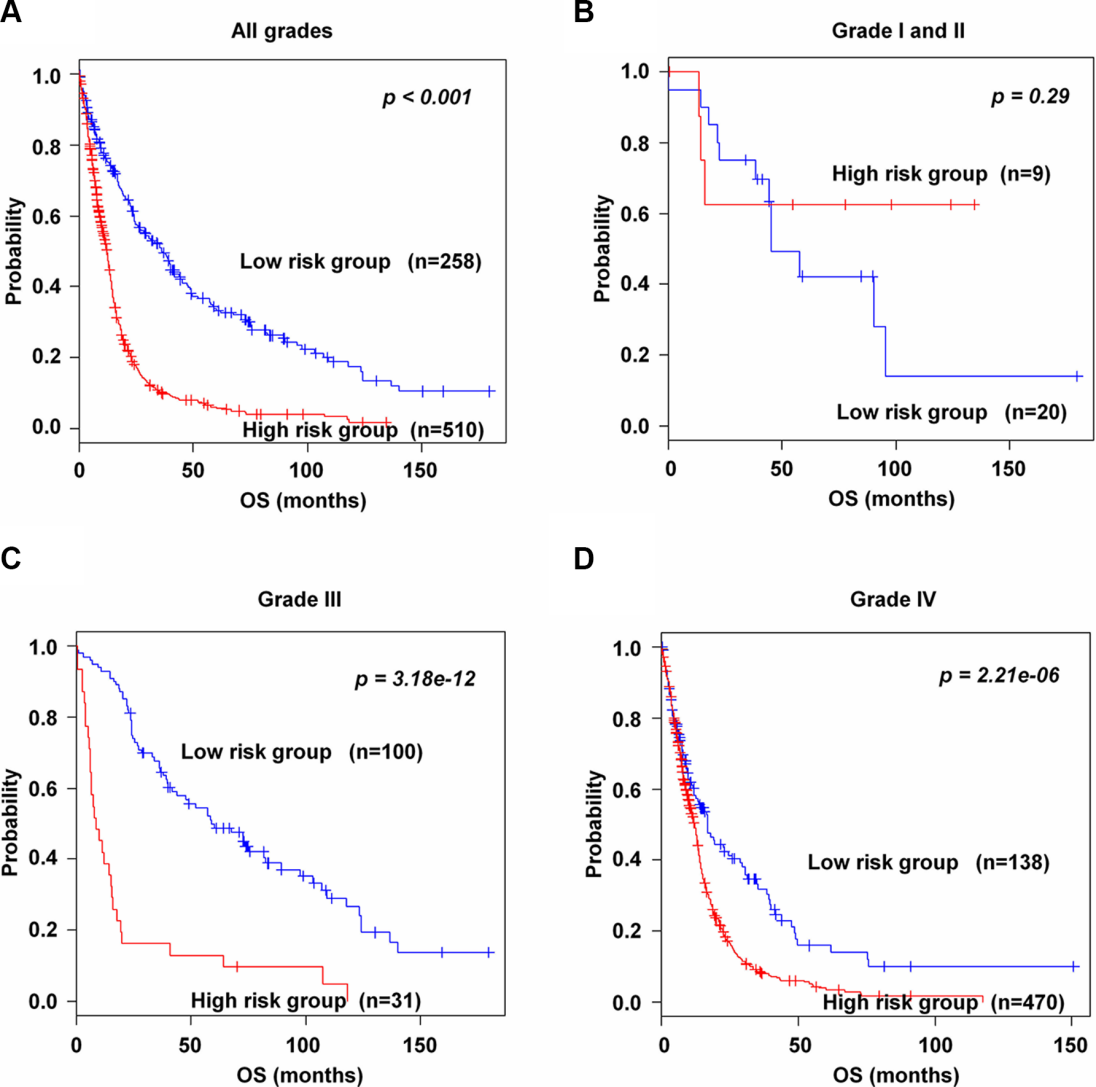
Kaplan-Meier survival analysis of the 89-gene signature in grades (**A**) Patients in all grades in the combined training and validation data sets. (**B**) Patients in grades I and II in the combined training and validation data sets. (**C–D**) Patients in grades III and IV in the combined training and validation data sets. Each group was classified into high and low risk groups. The *p* values were computed by the log-rank test.

To evaluate whether the 89-gene signature could classify patients by grade into high and low risk groups in the training and validation data sets, patients were combined in each grade; I (*n* = 6), II (*n* = 23), III (*n* = 131) and IV (*n* = 608). The 89-gene signature clearly stratified all combined patients into high and low risk groups (*p* < 0.001, Figure 5A). It could not significantly classify combined patients in grades I and II into two groups (*p* = 0.29, Figure 5B). However, the 89-gene signature significantly separated patients in grade III and IV into high and low risk groups (*p* = 3.18e–12 and *p* = 2.12e–06, Figure 5C–5D, respectively). Thirty one (23.6%) and 100 (76.3%) patients were classified into high and low risk in grade III, respectively. Four hundreds seventy (77.3%) and 138 (22.7%) patients were classified in grade IV into high and low risk. Similar results were obtained in patients in grade III and IV in training set (Supplementary Figure S6A–S6B, respectively) and all combined validation data sets (Supplementary Figure S6C–S6D, respectively).

### Association of the 89-gene signature with benefits of adjuvant chemotherapy and radiotherapy

**Figure 6:**
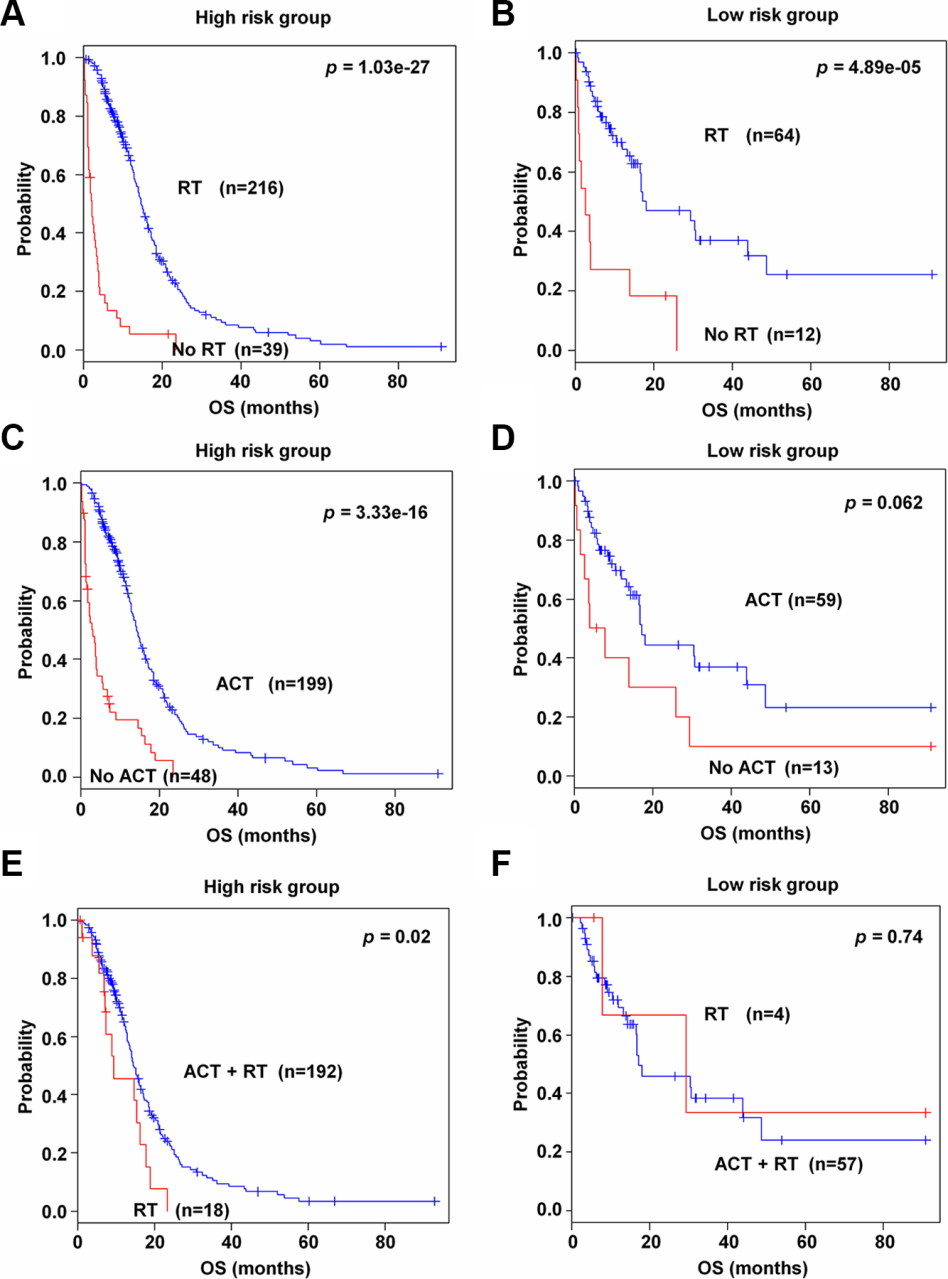
Kaplan-Meier survival analysis of the 89-gene signature with adjuvant chemotherapy and radiation therapy (**A–B**) Patients in high and low risk groups with radiotherapy in TCGA data set. (**C–D**) Patients in high and low risk groups with chemotherapy in TCGA data set. (**E–F**) Patients in high and low risk groups with combined therapies in TCGA data set. Each group was stratified according to chemotherapy, radiotherapy, and combined therapies. The *p* values were computed by the log-rank test.

To find the association of the 89-gene signature with response to chemotherapy and radiotherapy, subset analyses were performed in TCGA data set, for which therapeutic information were available. As shown in Figure 6A and 6B, patients in both high and low risk groups benefitted from radiation therapy (*p* = 1.03e–27 and *p* = 4.89e–05, respectively). By incorporating the 89-gene signature into chemotherapy information, only high risk group was shown to obtain benefit compared to patients without chemotherapy. In high risk group, over half of patients (80.5 %) benefited from chemotherapy (*p* = 3.33e–16, Figure 6C). On the contrary, low risk group did not have significant benefit from chemotherapy (*p* = 0.062, Figure 6D). Interestingly, high risk group had better response to combined therapies (*p* = 0.02, Figure 6E), while low risk groups did not get benefit from combined therapies (*p* = 0.74, Figure 6F). Additionally, with the EUMC data set, similar results were observed in chemotherapy and radiotherapy (Supplementary Figure S7A–S7F).

### Protein network and gene ontology in the 89-gene signature

The 89 probe sets corresponded to 89 annotated genes (Supplementary Table S1). So, we could clarify protein interactions of 89 genes in the 89-gene signature. To understand how these genes could be involved in networks related to glioma, we performed analysis using the STRING database that was able to elaborate physical and functional associations among proteins. The results showed that 81 out of 89 genes were closely connected in a single network (Supplementary Figure S8). We also investigated interactions using the Ingenuity Pathway Analysis (IPA) software and found putative networks related to NF-KB, STAT3, and AP-1 transcription factors (Supplementary Figure S9). In addition, 23 genes were involved in the activation of these transcription factors pathways. To identify the biological function of genes in the 89-gene signature, we performed GO enrichment analysis in DAVID, and then revealed 89 significant biological pathways involved in glioma. The top 61 important pathways with *p* < 0.05 were selected. They are involved in multiple cancer-related processes, such as cell-cell signaling, wound healing, inflammatory response (Supplementary Table S2).

## DISCUSSION

Several reports have been published to predict prognosis in gliomas [3, 13–15]. Studies based on gene expression profiles have been reported to classify patients according to known prognostic factors; however, no report has yet predicted chemotherapy response in gliomas. An unsupervised clustering approach was integrated to construct the 89-gene signature from the training set. The 89-gene signature was validated for its prognostic significance in three microarray independent data sets (TCGA, UCLA and MDAS) and one RNA-seq data (TCGA). Univariate and multivariate analyses showed significant association of the prognostic gene signature with survival after adjusting clinical covariates. In addition, the 89-gene signature has the ability to identify patients benefiting from chemo-and radiotherapy. Therefore, the established gene signature might be helpful in clinical management.

Tumor grade is the major clinical variable used to make glioma treatment decisions [3]. However, heterogeneity observed in therapeutic response among patients within the same histological grade indicates that histological classification is not an adequate predictor of the clinical behavior of a tumor [22]. Moreover, histological classification is based on subjective criteria, and lacks reproducibility [6]. In the present study, although patients in low risk group more often were presented with grade IV glioma than those in high risk group (Table 2), patients in high risk group showed poorer OS than those in low risk group. Considering the above results, we concluded that the 89-gene signature was more correlated with survival than histological classification, a finding which is also supported by previous reports [6, 14]. Additionally, the 89-gene signature stratified patients with grades III and IV glioma into high and low risk groups. Interestingly, patients with grades I and II glioma could not be stratified into two risk groups using this signature. Although we cannot definitively assert why the 89-gene signature could not stratify patients with grades I and II glioma, we hypothesize that this is due to the small number of patients analyzed in this study. Considering that grades III and IV gliomas are rapidly progressive malignant tumors [4], it is noteworthy that the 89-gene signature could stratify these patients even harboring tumor heterogeneity.

Recently described molecular markers, such as IDH1 mutation and 1p/19q co-deletion, are considered predictive of clinical outcomes for glioma patients [18, 23]. The IDH1 mutation is a strong predictor of outcome irrespective of histological type and grade [18, 19]. However, several studies have shown a higher rate of malignant transformation in IDH-mutated low grade glioma than in wild-type IDH1 tumors, showing that a subset of patients with the IDH1 mutation are characterized as having secondary glioblastoma [24, 25]. Additionally, oligodendrogliomas with 1p/19q co-deletion have been shown to progress more slowly and respond better to treatment [20, 26, 27]. However, the prognostic value of 1p/19q co-deletion in glioblastoma remains unknown [28]. Considering of diverse studies, molecular markers should be further defined according to molecular heterogeneity. In the present study, the 89-gene signature could stratify patients with IDH1 mutation and 1p/19q co-deletion status into high and low risk groups. In agreement with previous studies, most patients with the IDH1 mutation and 1p/19q co-deletion were classified as low risk, whereas most patients who had wild type for these markers were classified as high risk. However, it is meaningful that we could precisely predict the clinical behavior of tumors in individual patients within same molecular state. Additionally, these results suggest that the 89-gene signature has overcome the limitations of genetic molecular approaches in assessing tumor heterogeneity. By incorporating our gene signature into clinical information, patients could get more benefits in clinical practice.

Recent reports have shown that glioblastoma patients under the age of 40 years survive longer than those patients over 40 years [21]. In the present study, young age (< 40) was an independent significant prognostic factor. However, it remains unclear whether young age confers a favorable prognosis for children with glioblastoma [29]. One previous study reported that the prognosis was unfavorable for pediatric patients with glioblastoma. Another study reported favorable prognosis for glioblastoma patients over 70 years old, with a 2-year overall survival rate of 20% [30]. Considering the heterogeneity in prognosis within a single age group, more defined prognostic factors might be required to stratify such patients. Our study showed that the 89-gene signature could further stratify both young and old patients into high and low risk groups. As expected based on previous reports, most young patients (64.6%) were classified as low risk, whereas most old patients (75.5%) were classified as high risk, suggesting that the 89-gene signature could be of clinical value by subclassifying patients within same age group and so helping to make treatment plan decisions for individual patients.

Generally, adjuvant chemotherapy (ACT) and radiotherapy (RT) after surgery constitute the standard treatment in glioblastoma grade IV [31, 32]. However, addition of ACT to RT remains controversial in grade III anaplastic gliomas [3]. Unfortunately, no gene signatures related to ACT sensitivity in glioma have been discovered yet, although one prior study involving the EUMC data set with small number of patients who had ACT demonstrated that some genes were implicated in chemoradiation sensitivity [13]. Our subset analysis of patients with available chemotherapy information suggested that the 89-gene signature could predict patients who would benefit from ACT. Our study showed that patients in high risk group had significantly improved outcome with ACT, whereas patients in low risk group did not get significant benefit from ACT in all patients with available treatment data sets. Considering that high risk group carried a poorer prognosis than those in low risk group, our 89-gene signature has the potential to facilitate clinical decisions on using ACT for grade III glioma because of a poorer prognosis. The utility of the gene signature for treatment management in glioma still needs to be further evaluated in a prospective ACT clinical trial.

Most of the identified 89 genes play a critical role in aggressiveness, angiogenesis, local invasion, migration, and proliferation. These genes included COL1A2, COL3A1, CLO6A3 [33], FABP7 [16, 34–37], GDF-15, SH3GL2 [38, 39], ADM, VEGFA, and PTX3 [40, 41]. Our gene signature also contained proneural genes, BMP2, DCX, IGFBP2, PDPN, and PLAT, which are associated with anaplastic oligodendroglioma harboring 1p/19q co-deletion [42]. The proneural genes indicate a better prognosis of malignant glioma [43]. The 89-gene signature consists of a number of hypoxia and inflammation-related genes such as AKR1C3 [44], PTX3, PLAT [45] and IGFBP2 [46], showing that these two inseparable hallmarks are involved in tumor progression [47, 48] and play significant roles in glioma pathogenesis. Purinergic signaling related genes such as GPR17 [49], VEGFA [50] and CCL2 [51] are involved in inflammation leading to glioma growth [52, 53]. In addition, BCAT1 was reported to promote cell proliferation in gliomas carrying wild-type IDH1 [54]. Our gene signature also possessed several genes related to clinical characteristics of recurrent glioblastoma. Furthermore, genes such as AKR1C3, ETNPPL, FXYD1, SH3GL2, SH3GL3, SNAP91, and SYT1 have important role in reprograming and are also involved in drug resistance [55]. Additionally, IPA revealed that most of the genes were controlled by NF-KB, STAT3, and AP-1 transcription factors. These transcription factors play a vital role in cancer and are important regulators of immune and inflammatory functions [56–58]. Finally, many novel genes including CHRNA9, CNGA3, FERMT1, FZD7, GABRB3, METTL7B, USD5, and SYT4 were also identified, suggesting that our 89-gene signature contains novel information which may provide new biomarkers to assist in clinical decision making concerning new opportunities for targeted treatment of individual patients.

In conclusion, we identified the 89-gene signature as a highly discriminative predictor of prognosis. The prognostic value of the 89-gene signature was statistically significant in a reliable and reproducible manner across independent and combined data sets. Furthermore, our study revealed that patients could be stratified into high and low risk groups with different OS regardless of histology classification and molecular markers. In addition, the 89-gene signature might suggest which patients would benefit from ACT. Therefore, we propose that the 89-gene signature has an independent and accurate prognostic value for clinical use. Also, this study offers new opportunities for novel targeted treatment of individual patients.

## MATERIALS AND METHODS

### Patients and gene expression data

All gene expression data sets were obtained from the National Center for Biotechnology Information Gene Expression Omnibus database (http://www.ncbi.nlm.nih.gov/geo) and The Cancer Genome Atlas database (http://cancergenome.nih.gov/). Data were selected based on following criteria: raw CEL files and clinical information of patients with survival event and time. The raw data were preprocessed using robust multiarray averaging (RMA) method for normalization. Gene expression data from the GSE16011 (*n* = 264, Erasmus University Medical Center (EUMC)) was used as the training data set. The Cancer Genome Atlas data (TCGA, *n* = 342), GSE4412 (*n* = 85, University of California Los Angeles (UCLA)) and GSE4271 (*n* = 77, MD Anderson (MDAS)) were used as validation data sets (Table 1). To test the prognostic significance of gene expression signature, we used only gene expression data with available survival data. The information of adjuvant chemotherapy and radiotherapy were available for only 285 and 473 patients respectively from EUMC and TCGA data sets. In addition, RNA-seq data from TCGA (*n* = 165) was also used as validation data set (https://genome-cancer.ucsc.edu).

### Development of the prognostic gene expression signature

A gene expression signature was developed from the EUMC data set. Gene expression was generated by using the Affymetrix GeneChip Human Genome U133 Plus 2.0 chip set. Differentially expressed probe sets were identified among two classes using a random-variance*t* test. Differences of probe sets' expression between two classes were considered statistically significant if their *p* value was less than 0.001. A global and permutation tests were performed to investigate whether the expression profiles differed between the classes. The cluster analysis was performed with Cluster 3.0 (http://bonsai.hgc.jp/~mdehoon/software/cluster) and Tree View (http://www.eisenlab.org/eisen/). Although initially 129 probe sets were identified for constructing prediction models in t test analysis, only 89 probe sets, which were shared in both U133 Plus 2.0 and U133A, were used for all validation data sets (Supplementary Table S1).

### Validation of the prognostic gene expression signature

The validation of the gene signature was accomplished on independent data sets. Gene expression data from validation data sets were adjusted individually by subtracting the median expression value across the samples. To integrate each validation data set for constructing prediction models, 89 probe sets were aligned in each data set. To further refine this model and to sub-stratify the predicted outcomes, Compound Covariate Predictor (CCP) was utilized as a class prediction algorithm [59]. Gene expression data in the training set were combined to form a classifier according to CCP. The robustness of the classifier was estimated by the misclassification rate determined during the leave-one-out cross-validation (LOOCV) in the training set. During prediction, the cross-validation process omitted one sample at a time. For each sample omitted, the entire analysis was repeated from scratch, including the determination of genes which were univariately significant on the reduced training sample. From that gene list, a multivariate predictor was constructed and applied to the sample that was omitted. The program recorded whether that prediction was correct or not. This was repeated, omitting all of the samples one at a time.

Kaplan–Meier survival analyses were performed after the patient classification into two predicted subgroups, and Chi-square (*χ*^2^) and log-rank tests were used to evaluate the survival risk between two predicted subgroups of patients. The uni-and multi-variate Cox proportional hazards model were used to evaluate independent prognostic factors associated with survival, gene signature, tumor grade, age, and adjuvant chemotherapy as covariates.

### Pathway analysis

To perform pathway analysis on these expressed genes between subtypes, we used GO term enrichment analysis, using the Database for Annotation, Visualization and Integrated Discovery (DAVID) bioinformatics resource (http://david.abcc.ncifcrf.gov/home.jsp). Pathway analysis was performed to map genes to the Biological Process (BP) categories of GO and then calculate the significance of overrepresented categories in the selected gene list. The *p* value less than 0.05 was used to define significant pathways.

### Gene network analysis

Protein-protein interactions were predicted using the Search Tool for the Retrieval of Interacting Genes/Proteins (STRING) database v10.0 (http://www.string-db.org/). Proteins were linked based on the following six criteria; neighborhood, gene fusion, co-occurrence, co-expression, experimental evidence and existing databases [60]. Ingenuity Pathways Analysis (IPA) (http://www.ingenuity.com) was also used for gene network analysis, using a global molecular network developed from information contained in the Ingenuity Knowledge Database. Identified gene networks were ranked to score (z-score = 02).

### Statistical methods of microarray data

Microarray data and heatmap were analyzed using BRB-Array Tools Version 3.0 (http://linus.nci.nih.gov/BRB-ArrayTools.html). All other statistical analyses were accomplished in the R language environment (http://www.r-project.org) and Statistical Package for Social Sciences (SPSS) software (version 20, SPSS Inc, Chicago, IL, USA). In all statistical analyses, *p* value of less than 0.05 was considered significant.

## SUPPLEMENTARY MATERIALS FIGURES




