# Supplementary material for: Prognostic value and their clinical implication of 89-gene signature in glioma
Source: Oncotarget. 2016 Jun 13;7(32):51237–50. doi: 10.18632/oncotarget.9983 (PMC5239472; doi:10.18632/oncotarget.9983)
Supplement: Supplementary file 1 [file oncotarget-07-51237-s001.pdf]

Prognostic value and their clinical implication of 89-gene signature in glioma

Supplementary Materials

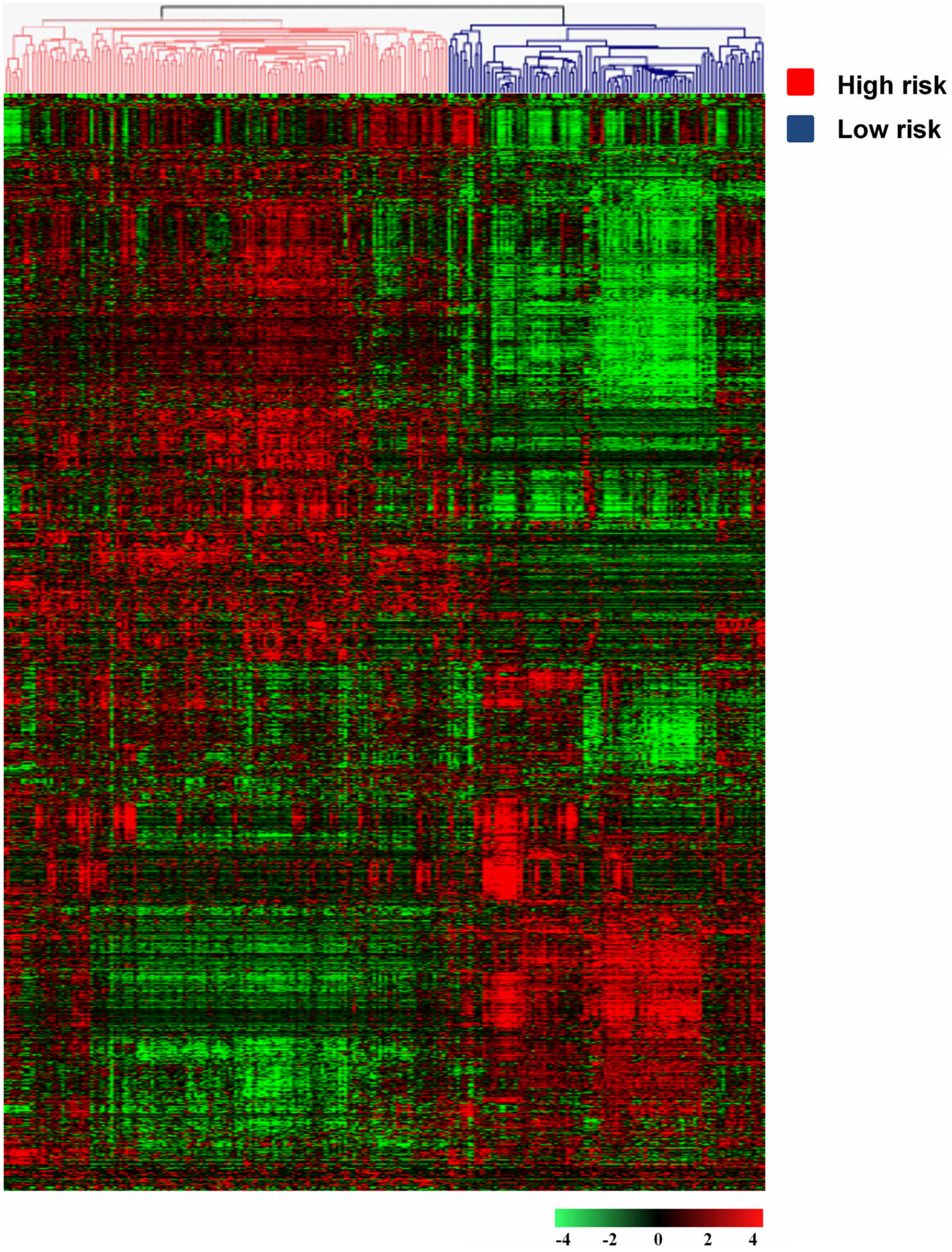

Supplementary Figure S1: Hierarchical clustering analysis of gene expression data from the training set. Genes with an expression level that had at least 2-fold difference relative to median value across 30 samples were selected for hierarchical clustering analysis (1,009 gene features). Expression profiles (red, relative high expression; green, relative low expression) between high and low risk groups in the training set.

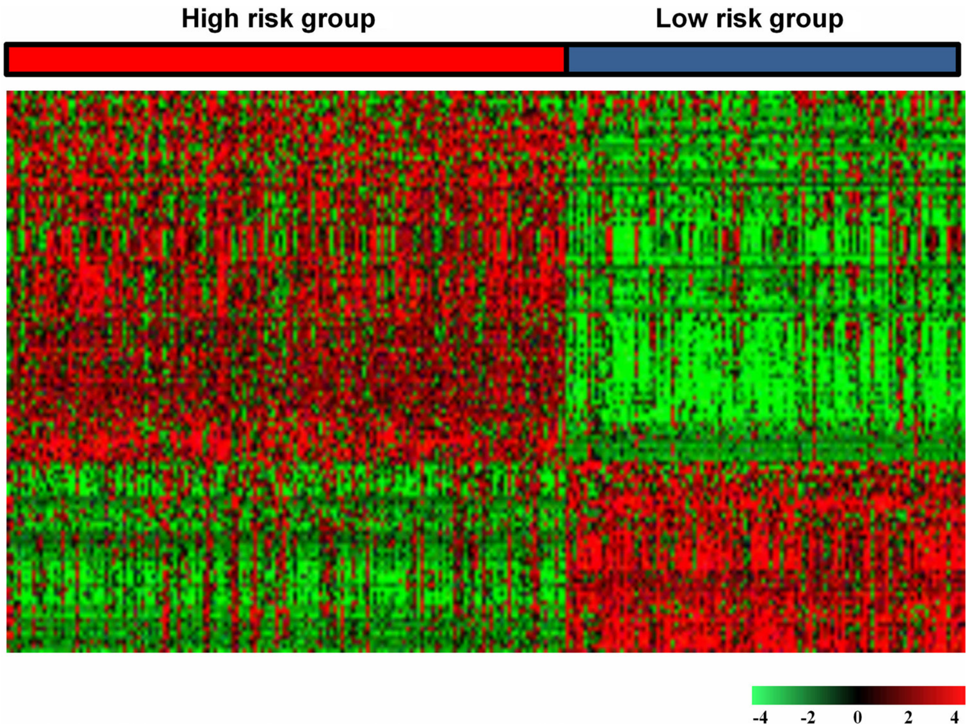

Supplementary Figure S2: Differentially expressed genes between high and low risk groups in the training set. Genes were selected by two-sample t-test with permutation test and stringent cut-off ($p < 0.001$ and 2.5-fold difference) was applied to retain genes whose expression was significantly between the two groups (129 genes). Expression profiles (red, relative high expression; green, relative low expression) between high and low risk groups in the training set.

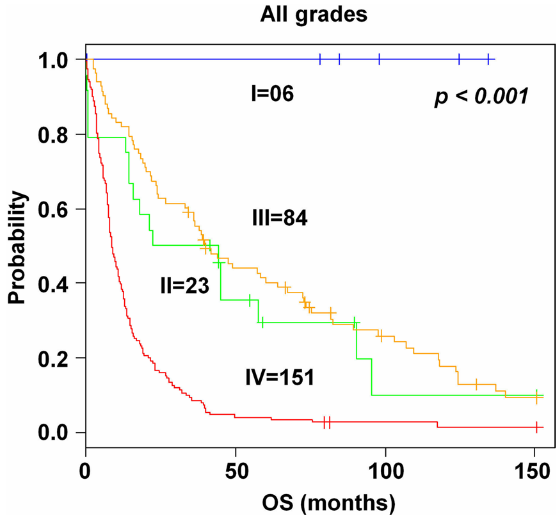

Supplementary Figure S3: Kaplan-Meier survival analysis of histology grades in the training set. Patients showed a significant difference according to the histology grades in the training set. The *p* values were computed by the log-rank test.

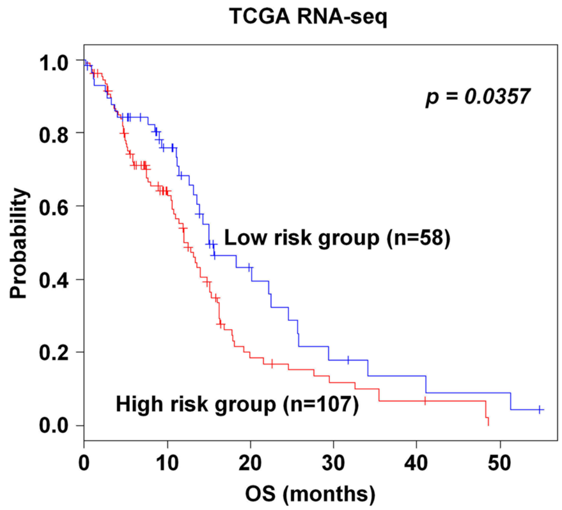

Supplementary Figure S4: Prognostic significance of the 89-gene signature in RNA-seq TCGA data set. Kaplan-Meier survival plots of overall survival (OS) of the two groups in the RNA-seq data. The *p* values were computed by the log-rank test.

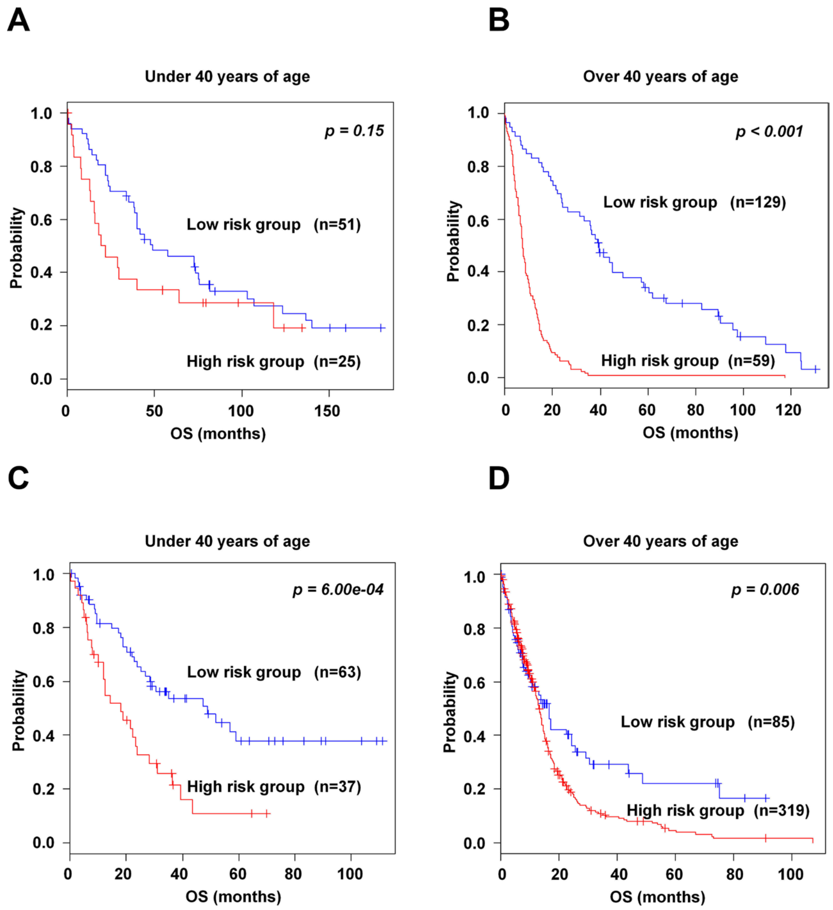

Supplementary Figure S5: Kaplan-Meier survival analysis of age in the training and validation data sets. (**A–B**) Patients under and over 40 years of age groups in the training data set. (**C–D**) Patients under and over 40 years of age groups in the validation data sets. Each group was classified by the 89-gene signature into high and low risk groups. The *p* values were computed by the log-rank test.

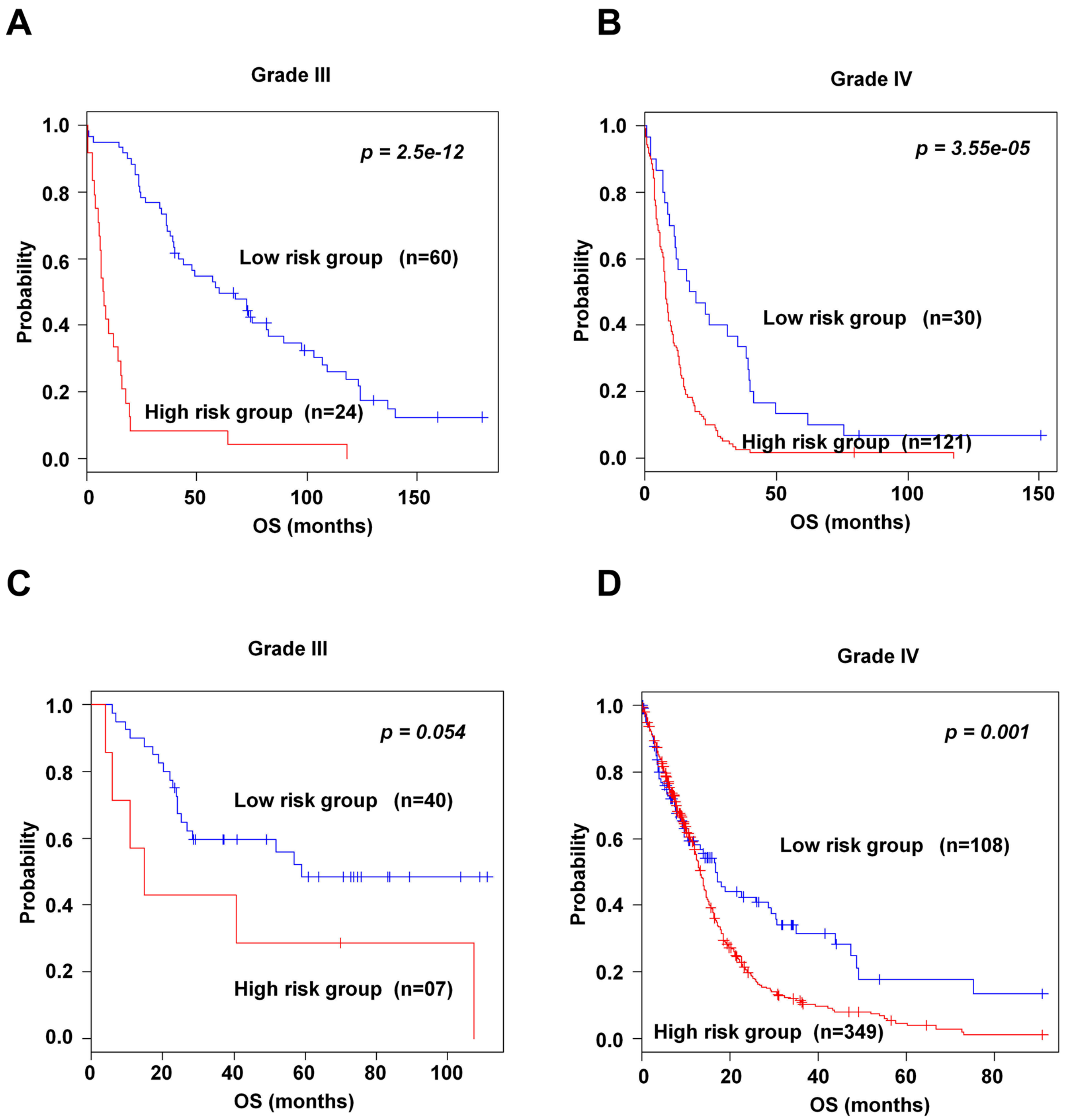

Supplementary Figure S6: Kaplan-Meier survival analysis of grades III and IV in the training and validation data sets. (**A–B**) Patients in grades III and IV in the training data set. (**C–D**) Patients in grades III and IV in the validation data sets. Each group was classified by the 89-gene signature into high and low risk groups. The *p* values were computed by the log-rank test.

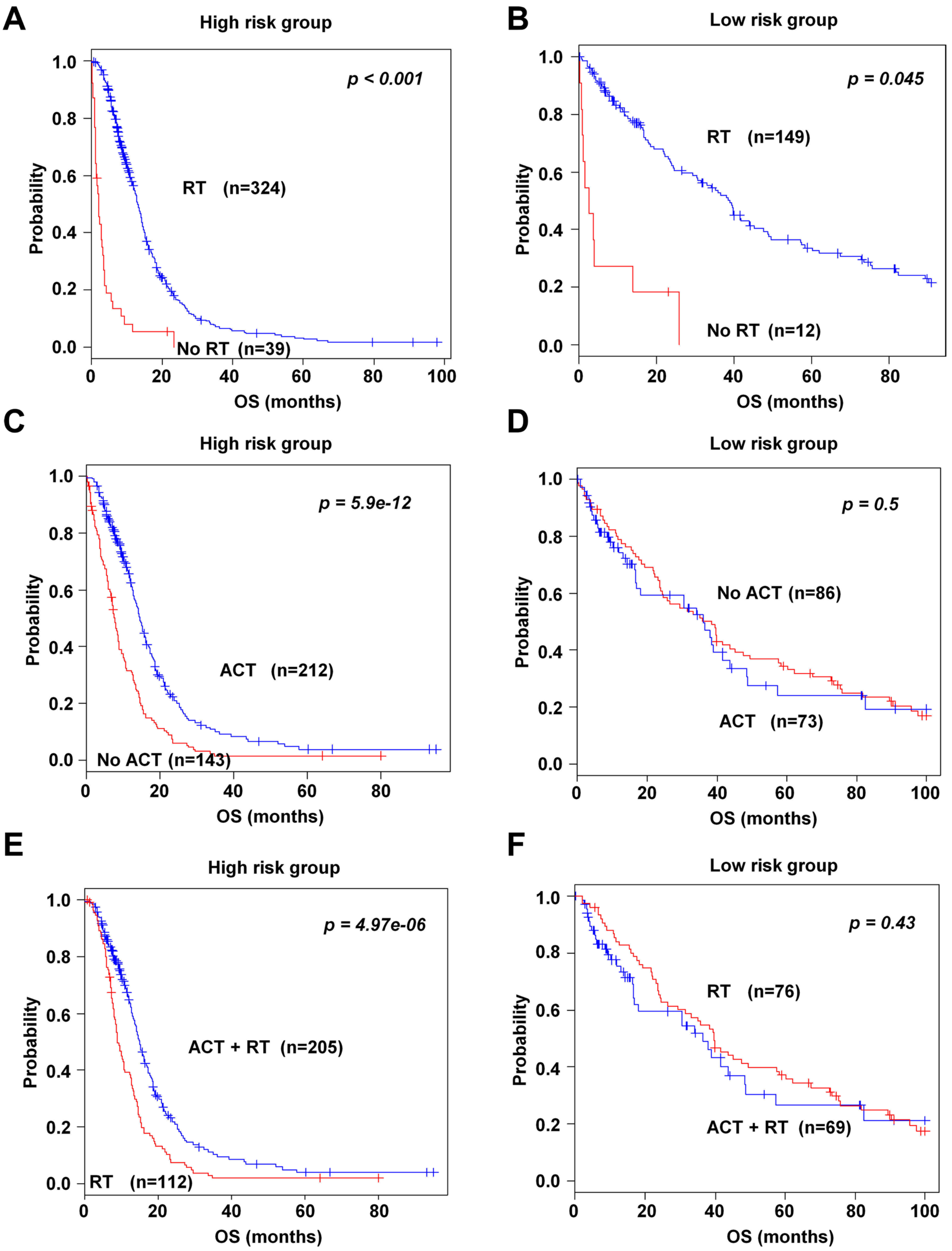

Supplementary Figure S7: Kaplan-Meier survival analysis of adjuvant chemotherapy and radiation therapy in the training by EUMC and validation by TCGA data sets. (**A–B**) Patients with radiotherapy in the EUMC and TCGA data sets. (**C–D**) Patients with chemotherapy in the EUMC and TCGA data sets. (**E–F**) Patients with combined therapies in the EUMC and TCGA data sets. Each group was stratified according to chemotherapy, radiotherapy, and combined therapies. The *p* values were computed by the log-rank test.

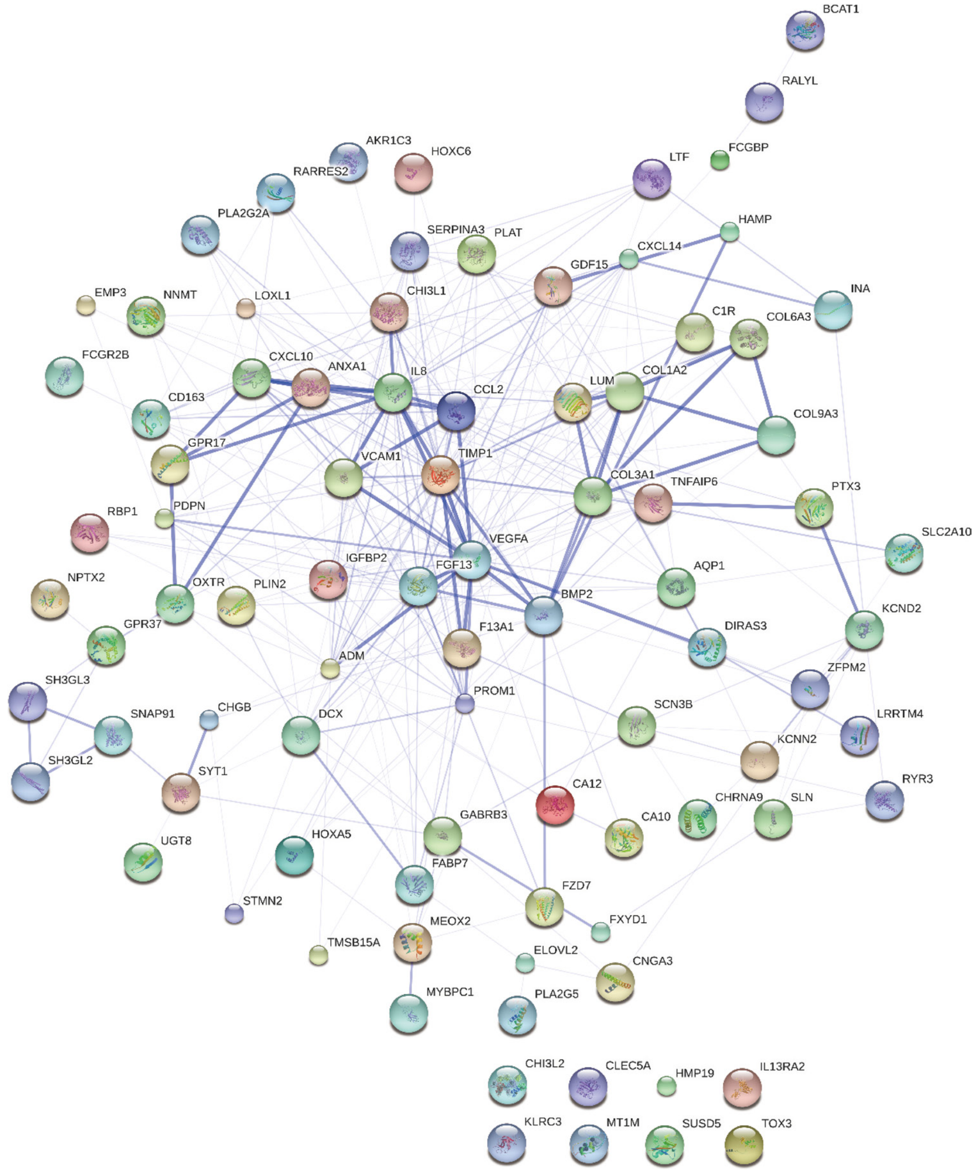

Supplementary Figure S8: Protein interaction network analysis in the 89-gene signature. Interaction map was generated using the STRING database.

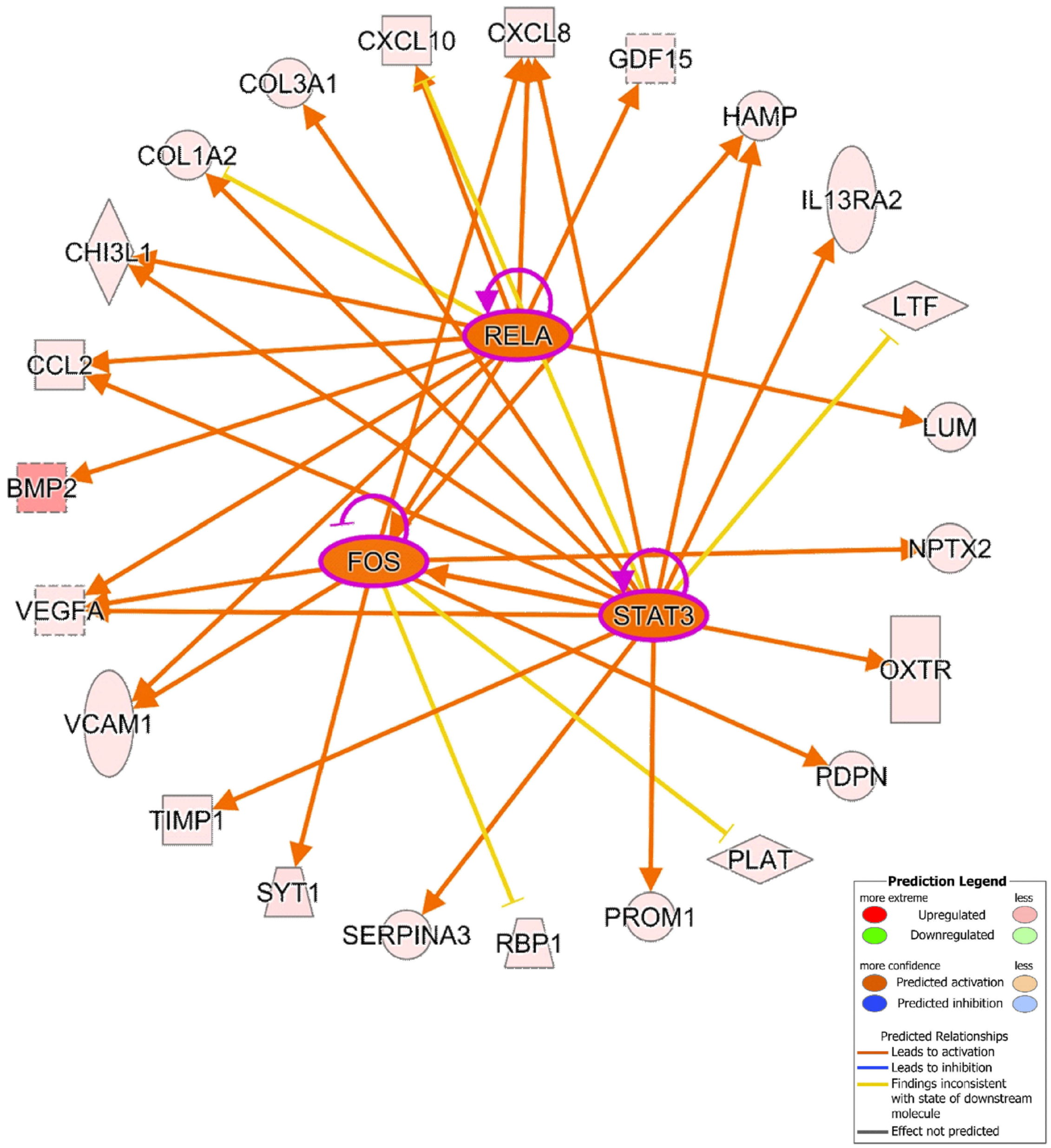

Supplementary Figure S9: NF-KB, AP-1, and STAT3 networks from Ingenuity pathway analysis. Gene networks from IPA showed upstream genes significantly associated with NF-KB, AP-1, and STAT3 pathways.

Supplementary Table S1: Annotation of the 89-gene signature. See Supplementary_Table_S1 and S2

Supplementary Table S2: Gene ontology (GO) analysis of statistically significant genes in the 89-gene signature in glioma. See Supplementary_Table_S1 and S2
