# Supplementary material for: Prognostic value and their clinical implication of 89-gene signature in glioma
Source: Oncotarget. 2016 Jun 13;7(32):51237–50. doi: 10.18632/oncotarget.9983 (PMC5239472; doi:10.18632/oncotarget.9983)
Supplement: Supplementary file 2 [file oncotarget-07-51237-s002.docx]

**Supplementary Table S1:** Annotation of the 89-gene signature

| ProbeSet | Name | Accession | Symbol |
| --- | --- | --- | --- |
| 221576_at | growth differentiation factor 15 | BC000529 | GDF15 |
| 221107_at | cholinergic receptor, nicotinic, alpha 9 (neuronal) | NM_017581 | CHRNA9 |
| 221024_s_at | solute carrier family 2 (facilitated glucose transporter), member 10 | NM_030777 | SLC2A10 |
| 221008_s_at | ethanolamine-phosphate phospho-lyase | NM_031279 | ETNPPL |
| 220889_s_at | carbonic anhydrase X | NM_020178 | CA10 |
| 220491_at | hepcidin antimicrobial peptide | NM_021175 | HAMP |
| 220345_at | leucine rich repeat transmembrane neuronal 4 | NM_024993 | LRRTM4 |
| 220116_at | potassium intermediate/small conductance calcium-activated channel, subfamily N, member 2 | NM_021614 | KCNN2 |
| 219890_at | C-type lectin domain family 5, member A | NM_013252 | CLEC5A |
| 219778_at | zinc finger protein, FOG family member 2 | NM_012082 | ZFPM2 |
| 218623_at | HMP19 protein | NM_015980 | HMP19 |
| 218002_s_at | chemokine (C-X-C motif) ligand 14 | NM_004887 | CXCL14 |
| 217546_at | metallothionein 1M | R06655 | MT1M |
| 216598_s_at | chemokine (C-C motif) ligand 2 | S69738 | CCL2 |
| 215506_s_at | DIRAS family, GTP-binding RAS-like 3 | AK021882 | DIRAS3 |
| 214954_at | sushi domain containing 5 | BF977837 | SUSD5 |
| 214774_x_at | TOX high mobility group box family member 3 | AK027006 | TOX3 |
| 214390_s_at | branched chain amino-acid transaminase 1, cytosolic | AI652662 | BCAT1 |
| 214087_s_at | myosin binding protein C, slow type | BF593509 | MYBPC1 |
| 213967_at | RALY RNA binding protein-like | AI634532 | RALYL |
| 213844_at | homeobox A5 | NM_019102 | HOXA5 |
| 213712_at | ELOVL fatty acid elongase 2 | BF508639 | ELOVL2 |
| 213479_at | neuronal pentraxin II | U26662 | NPTX2 |
| 213060_s_at | chitinase 3-like 2 | U58515 | CHI3L2 |
| 212067_s_at | complement component 1, r subcomponent | AL573058 | C1R |
| 210889_s_at | Fc fragment of IgG, low affinity IIb, receptor (CD32) | M31933 | FCGR2B |
| 210512_s_at | vascular endothelial growth factor A | AF022375 | VEGFA |
| 209631_s_at | G protein-coupled receptor 37 | U87460 | GPR37 |
| 209496_at | retinoic acid receptor responder (tazarotene induced) 2 | BC000069 | RARRES2 |
| 209395_at | chitinase 3-like 1 (cartilage glycoprotein-39) | M80927 | CHI3L1 |
| 209160_at | aldo-keto reductase family 1, member C3 | AB018580 | AKR1C3 |
| 209122_at | perilipin 2 | BC005127 | PLIN2 |
| 208358_s_at | UDP glycosyltransferase 8 | NM_003360 | UGT8 |
| 207723_s_at | killer cell lectin-like receptor subfamily C, member 3 | NM_002261 | KLRC3 |
| 207542_s_at | aquaporin 1 (Colton blood group) | NM_000385 | AQP1 |
| 207261_at | cyclic nucleotide gated channel alpha 3 | NM_001298 | CNGA3 |
| 207103_at | potassium voltage-gated channel, Shal-related subfamily, member 2 | NM_012281 | KCND2 |
| 206858_s_at | homeobox C6 | NM_004503 | HOXC6 |
| 206825_at | oxytocin receptor | NM_000916 | OXTR |
| 206306_at | ryanodine receptor 3 | NM_001036 | RYR3 |
| 206201_s_at | mesenchyme homeobox 2 | NM_005924 | MEOX2 |
| 206190_at | G protein-coupled receptor 17 | NM_005291 | GPR17 |
| 206178_at | phospholipase A2, group V | NM_000929 | PLA2G5 |
| 206172_at | interleukin 13 receptor, alpha 2 | NM_000640 | IL13RA2 |
| 206157_at | pentraxin 3, long | NM_002852 | PTX3 |
| 206025_s_at | tumor necrosis factor, alpha-induced protein 6 | AW188198 | TNFAIP6 |
| 205850_s_at | gamma-aminobutyric acid (GABA) A receptor, beta 3 | NM_000814 | GABRB3 |
| 205751_at | SH3-domain GRB2-like 2 | NM_003026 | SH3GL2 |
| 205636_at | SH3-domain GRB2-like 3 | AF036269 | SH3GL3 |
| 205384_at | FXYD domain containing ion transport regulator 1 | NM_005031 | FXYD1 |
| 205374_at | sarcolipin | NM_003063 | SLN |
| 205347_s_at | thymosin beta 15a | NM_021992 | TMSB15A |
| 205289_at | bone morphogenetic protein 2 | AA583044 | BMP2 |
| 205110_s_at | fibroblast growth factor 13 | NM_004114 | FGF13 |
| 205029_s_at | fatty acid binding protein 7, brain | NM_001446 | FABP7 |
| 204953_at | synaptosomal-associated protein, 91kDa | NM_014841 | SNAP91 |
| 204879_at | podoplanin | NM_006474 | PDPN |
| 204850_s_at | doublecortin | NM_000555 | DCX |
| 204724_s_at | collagen, type IX, alpha 3 | NM_001853 | COL9A3 |
| 204722_at | sodium channel, voltage-gated, type III, beta subunit | AW007335 | SCN3B |
| 204533_at | chemokine (C-X-C motif) ligand 10 | NM_001565 | CXCL10 |
| 204465_s_at | internexin neuronal intermediate filament protein, alpha | NM_004692 | INA |
| 204304_s_at | prominin 1 | NM_006017 | PROM1 |
| 204260_at | chromogranin B (secretogranin 1) | NM_001819 | CHGB |
| 203998_s_at | synaptotagmin I | AV723167 | SYT1 |
| 203963_at | carbonic anhydrase XII | NM_001218 | CA12 |
| 203868_s_at | vascular cell adhesion molecule 1 | NM_001078 | VCAM1 |
| 203729_at | epithelial membrane protein 3 | NM_001425 | EMP3 |
| 203705_s_at | frizzled class receptor 7 | AI333651 | FZD7 |
| 203649_s_at | phospholipase A2, group IIA (platelets, synovial fluid) | NM_000300 | PLA2G2A |
| 203645_s_at | CD163 molecule | NM_004244 | CD163 |
| 203570_at | lysyl oxidase-like 1 | NM_005576 | LOXL1 |
| 203423_at | retinol binding protein 1, cellular | NM_002899 | RBP1 |
| 203305_at | coagulation factor XIII, A1 polypeptide | NM_000129 | F13A1 |
| 203240_at | Fc fragment of IgG binding protein | NM_003890 | FCGBP |
| 203000_at | stathmin 2 | BF967657 | STMN2 |
| 202912_at | adrenomedullin | NM_001124 | ADM |
| 202859_x_at | chemokine (C-X-C motif) ligand 8 | NM_000584 | CXCL8 |
| 202718_at | insulin-like growth factor binding protein 2, 36kDa | NM_000597 | IGFBP2 |
| 202403_s_at | collagen, type I, alpha 2 | AA788711 | COL1A2 |
| 202376_at | serpin peptidase inhibitor, clade A (alpha-1 antiproteinase, antitrypsin), member 3 | NM_001085 | SERPINA3 |
| 202237_at | nicotinamide N-methyltransferase | NM_006169 | NNMT |
| 202018_s_at | lactotransferrin | NM_002343 | LTF |
| 201860_s_at | plasminogen activator, tissue | NM_000930 | PLAT |
| 201852_x_at | collagen, type III, alpha 1 | AI813758 | COL3A1 |
| 201744_s_at | lumican | NM_002345 | LUM |
| 201666_at | TIMP metallopeptidase inhibitor 1 | NM_003254 | TIMP1 |
| 201438_at | collagen, type VI, alpha 3 | NM_004369 | COL6A3 |
| 201012_at | annexin A1 | NM_000700 | ANXA1 |

**Supplementary Table S2:** Gene ontology (GO) analysis of statistically significant genes in 89 gene signature in Gliomas

| GO term | Biological Process | p value | Genes symbol | count |
| --- | --- | --- | --- | --- |
| GO:0009611 | response to wounding | 1.24E-06 | PLAT, INA, BMP2, CCL2, PDPN, F13A1, COL3A1, ANXA1, C1R, CXCL10, CD163, TNFAIP6, ADM, SERPINA3, PTX3 | 15 |
| GO:0006952 | defense response | 7.01E-06 | BMP2, CCL2, KLRC3, PDPN, ANXA1, C1R, CD163, CXCL10, TNFAIP6, HAMP, SERPINA3, PLA2G2A, LTF, PTX3, CLEC5A | 15 |
| GO:0006954 | inflammatory response | 8.26E-05 | TNFAIP6, BMP2, CCL2, PDPN, ANXA1, SERPINA3, C1R, PTX3, CD163, CXCL10 | 10 |
| GO:0007267 | cell-cell signaling | 1.19E-04 | PLAT, SYT1, TNFAIP6, BMP2, KCND2, GABRB3, CXCL14, ADM, NPTX2, OXTR, FGF13, GDF15, CXCL10 | 13 |
| GO:007048 | response to oxygen levels | 0.00116927 | PLAT, CCL2, ADM, PDPN, VEGFA, OXTR | 6 |
| GO:0030005 | cellular di-, tri-valent inorganic cation homeostasis | 0.001717341 | FXYD1, CCL2, ADM, HAMP, RYR3, OXTR, LTF | 7 |
| GO:0055066 | di-, tri-valent inorganic cation homeostasis | 0.002226332 | FXYD1, CCL2, ADM, HAMP, RYR3, OXTR, LTF | 7 |
| GO:0007178 | transmembrane receptor protein serine/threonine kinase signaling pathway | 0.00267201 | BMP2, CCL2, COL3A1, COL1A2, GDF15 | 5 |
| GO:0001944 | vasculature development | 0.002841974 | PLAT, MEOX2, PDPN, VEGFA, COL3A1, COL1A2, ZFPM2 | 7 |
| GO:0030003 | cellular cation homeostasis | 0.003014093 | FXYD1, CCL2, ADM, HAMP, RYR3, OXTR, LTF | 7 |
| GO:0009612 | response to mechanical stimulus | 0.003814681 | BMP2, CCL2, CHRNA9, IGFBP2 | 4 |
| GO:0003013 | circulatory system process | 0.003911031 | MEOX2, ADM, VEGFA, COL1A2, OXTR, CXCL10 | 6 |
| GO:0008015 | blood circulation | 0.003911031 | MEOX2, ADM, VEGFA, COL1A2, OXTR, CXCL10 | 6 |
| GO:0048545 | response to steroid hormone stimulus | 0.004474128 | CCL2, ADM, OXTR, AQP1, CNGA3, IGFBP2 | 6 |
| GO:0046717  GO:0007179 | acid secretion  transforming growth factor beta receptor signaling pathway | 0.004495914  0.004634291 | ANXA1, OXTR, PLA2G5  CCL2, COL3A1, COL1A2, GDF15 | 3  4 |
| GO:0006873 | cellular ion homeostasis | 0.004965827 | FXYD1, KCND2, CCL2, ADM, HAMP, RYR3, OXTR, LTF | 8 |
| GO:0055080 | cation homeostasis | 0.005372235 | FXYD1, CCL2, ADM, HAMP, RYR3, OXTR, LTF | 7 |
| GO:0055082 | cellular chemical homeostasis | 0.00540995 | FXYD1, KCND2, CCL2, ADM, HAMP, RYR3, OXTR, LTF | 8 |
| GO:0015909 | long-chain fatty acid transport | 0.00610405 | PLIN2, ANXA1, PLA2G5 | 3 |
| GO:0048878 | chemical homeostasis | 0.007869225 | FXYD1, KCND2, CCL2, ADM, HAMP, RYR3, VEGFA, OXTR, LTF | 9 |
| GO:0050801 | ion homeostasis | 0.007990986 | FXYD1, KCND2, CCL2, ADM, HAMP, RYR3, OXTR, LTF | 8 |
| GO:0042592 | homeostatic process | 0.009036635 | FXYD1, KCND2, CCL2, ADM, HAMP, RYR3, VEGFA, SERPINA3, OXTR, LTF, TIMP1 | 11 |
| GO:0018149 | peptide cross-linking | 0.009277171 | F13A1, COL3A1, ANXA1 | 3 |
| GO:0006811 | ion transport | 0.010484823 | FXYD1, KCND2, SLN, GABRB3, CHRNA9, SCN3B, RYR3, KCNN2, LTF, AQP1, CNGA3 | 11 |
| GO:0015908 | fatty acid transport | 0.01071228 | PLIN2, ANXA1, PLA2G5 | 3 |
| GO:0030199 | collagen fibril organization | 0.011464263 | LUM, COL3A1, COL1A2 | 3 |
| GO:0031960 | response to corticosteroid stimulus | 0.012105053 | CCL2, ADM, CNGA3, IGFBP2 | 4 |
| GO:0001568 | blood vessel development | 0.012183252 | PLAT, MEOX2, VEGFA, COL3A1, COL1A2, ZFPM2 | 6 |
| GO:0042127  GO:0007167 | regulation of cell proliferation  enzyme linked receptor protein signaling pathway | 0.012307464  0.012395167 | VCAM1, BMP2, CCL2, ADM, VEGFA, ANXA1, PLA2G2A, EMP3, FABP7, TIMP1, CXCL10  PLAT, BMP2, CCL2, VEGFA, COL3A1, COL1A2, GDF15 | 11  7 |
| GO:0006955 | immune response | 0.014908728 | CCL2, CXCL14, FCGR2B, HAMP, VEGFA, LTF, C1R, PTX3, CLEC5A, CXCL10 | 10 |
| GO:0019725 | cellular homeostasis | 0.015571955 | FXYD1, KCND2, CCL2, ADM, HAMP, RYR3, OXTR, LTF | 8 |
| GO:0060137 | maternal process involved in parturition | 0.016760657 | CCL2, OXTR | 2 |
| GO:0030324 | lung development | 0.018182557 | PDPN, HOXA5, VEGFA, ZFPM2 | 4 |
| GO:0040007 | growth | 0.019542646 | INA, BMP2, HOXA5, DCX, EMP3 | 5 |
| GO:0006874 | cellular calcium ion homeostasis | 0.019542646 | FXYD1, CCL2, ADM, RYR3, OXTR | 5 |
| GO:0030323 | respiratory tube development | 0.019668689 | PDPN, HOXA5, VEGFA, ZFPM2 | 4 |
| GO:0055074 | calcium ion homeostasis | 0.02133809 | FXYD1, CCL2, ADM, RYR3, OXTR | 5 |
| GO:0060541 | respiratory system development | 0.022837193 | PDPN, HOXA5, VEGFA, ZFPM2 | 4 |
| GO:0009617 | response to bacterium | 0.023231963 | CCL2, ADM, HAMP, PLA2G2A, LTF | 5 |
| GO:0006875 | cellular metal ion homeostasis | 0.024416031 | FXYD1, CCL2, ADM, RYR3, OXTR | 5 |
| GO:0007268 | synaptic transmission | 0.026032333 | PLAT, SYT1, KCND2, GABRB3, NPTX2, OXTR | 6 |
| GO:0046903 | secretion | 0.026694598 | SYT1, ANXA1, OXTR, AQP1, PLA2G5, CXCL10 | 6 |
| GO:0055065 | metal ion homeostasis | 0.028185632 | FXYD1, CCL2, ADM, RYR3, OXTR | 5 |
| GO:0006690 | icosanoid metabolic process | 0.028608707 | AKR1C3, PDPN, PLA2G5 | 3 |
| GO:0015718 | monocarboxylic acid transport | 0.03207291 | PLIN2, ANXA1, PLA2G5 | 3 |
| GO:0007507 | heart development | 0.032761255 | BMP2, ADM, COL3A1, OXTR, ZFPM2 | 5 |
| GO:0033559 | unsaturated fatty acid metabolic process | 0.033262666 | AKR1C3, PDPN, PLA2G5 | 3 |
| GO:0035239 | tube morphogenesis | 0.03459575 | BMP2, PDPN, HOXA5, VEGFA | 4 |
| GO:0035295 | tube development | 0.035203478 | BMP2, PDPN, HOXA5, VEGFA, ZFPM2 | 5 |
| GO:0006812 | cation transport | 0.035405389 | KCND2, SLN, CHRNA9, SCN3B, RYR3, KCNN2, LTF, AQP1 | 8 |
| GO:0006032 | chitin catabolic process | 0.038677653 | CHI3L1, CHI3L2 | 2 |
| GO:0006030 | chitin metabolic process | 0.038677653 | CHI3L1, CHI3L2 | 2 |
| GO:0001666 | response to hypoxia | 0.039582383 | PLAT, CCL2, ADM, VEGFA | 4 |
| GO:0007155 | cell adhesion | 0.04219533 | VCAM1, TNFAIP6, CCL2, MYBPC1, PDPN, COL6A3, COL3A1, SUSD5, FCGBP | 9 |
| GO:0022610 | biological adhesion | 0.042493394 | VCAM1, TNFAIP6, CCL2, MYBPC1, PDPN, COL6A3, COL3A1, SUSD5, FCGBP | 9 |
| GO:0030001 | metal ion transport | 0.046367772 | KCND2, SLN, CHRNA9, SCN3B, RYR3, KCNN2, LTF | 7 |
| GO:0019226 | transmission of nerve impulse | 0.046849907 | PLAT, SYT1, KCND2, GABRB3, NPTX2, OXTR | 6 |
| GO:0010033 | response to organic substance | 0.048753676 | CCL2, PLIN2, ADM, COL3A1, OXTR, AQP1, CNGA3, IGFBP2, PLA2G5 | 9 |
| GO:0046942 | carboxylic acid transport | 0.049755827 | PLIN2, PDPN, ANXA1, PLA2G5 | 4 |

**Figure Legends**

**Figure 1:** **Survival analysis of the training data set.**

(A) The heatmap of the median centered 89 genes’ expression profiles (red, relative high expression; green, relative low expression) between high and low risk groups in the training data set.

(B) Kaplan-Meier plots of overall survival (OS) of the two groups in the training data set. The *p* values were computed by the log-rank test.

**Figure 2:** Prognostic significance of the 89-gene signature in independent validation data sets.

(A) Schematic overview of the strategy used for the construction of the prediction model and evaluation of predicted outcomes in three independent data sets by the 89-gene signature.

(B) All combined validation data sets were stratified by the 89-gene signature into two groups. The *p* values were computed by the log-rank test.

(C-E) Kaplan-Meier survival plots of overall survival (OS) of the two groups in three independent data sets: TCGA, UCLA, and MDAS.

**Figure 3:** Significant association of the 89-gene signature with molecular pathways and mutation **in the training data set.**

(A-B) Kaplan-Meier curves of patients in 1p/19q co-deletion and wild type groups.

(C-D) IDH1 mutation and wild type groups in the training data set. Patients were classified by the 89-gene signature. The *p* values were computed by the log-rank test.

**Figure 4:** Kaplan-Meier survival analysis of the 89-gene signature in age.

**(**A) The patients under 40 years of age group in the combined training and validation data sets were stratified into high and low risk groups.

(B) The patients over 40 years of age group in the combined training and validation data sets were stratified into high and low risk groups. The *p* values were computed by the log-rank test.

**Figure 5:** Kaplan-Meier survival analysis of the 89-gene signature in **grades.**

(A) Patients in all grades in the combined training and validation data sets.

(B) Patients in grades I and II patients in the combined training and validation data sets.

(C-D) Patients in grades III and IV patients in the combined training and validation data sets. Each group was classified into high and low risk groups. The *p* values were computed by the log-rank test.

**Figure 6:** Kaplan-Meier survival analysis of the 89-gene signature **with adjuvant chemotherapy and radiation therapy.**

(A-B) Patients in high and low risk groups with radiotherapy in the TCGA data set.

(C-D) Patients in high and low risk groups with chemotherapy in the TCGA data set.

(E-F) Patients in high and low risk groups with combined therapies in the TCGA data set. Each group was stratified according to chemotherapy, radiotherapy, and combined therapies. The *p* values were computed by the log-rank test.

**Supplementary Figure S1. Hierarchical clustering analysis of gene expression data from training set.**

Genes with an expression level that had at least 2-fold difference relative to median value across 30 samples were selected for hierarchical clustering analysis (1,009 gene features). Expression profiles (red, relative high expression; green, relative low expression) between high and low risk groups in the training set.

**Supplementary Figure S2. Differentially expressed genes between high and low risk groups in the training set.**

Genes were selected by two-sample t-test with permutation test and stringent cut-off (p < 0.001 and 2.5-fold difference) was applied to retain genes whose expression was significantly between the two groups (129 genes). Expression profiles (red, relative high expression; green, relative low expression) between high and low risk groups in the training set.

**Supplementary Figure S3.** Kaplan-Meier survival analysis of **histology grades** **in the training set.**

The patients showed a significant difference according to the histology grades in the training set. The *p* values were computed by the log-rank test.

**Supplementary Figure S4.** Prognostic significance of the 89-gene signature in RNA-seq TCGA data set.

Kaplan-Meier survival plots of overall survival (OS) of the two groups in the RNA-seq data. The *p* values were computed by the log-rank test.

**Supplementary Figure S5.** Kaplan-Meier survival analysis of age **in the training and validation data sets.**

(A-B) The patients under and over 40 years of age groups in the training data set.

(C-D) The patients under and over 40 years of age groups in the validation data sets. Each group was classified by the 89-gene signature into high and low risk groups. The *p* values were computed by the log-rank test.

**Supplementary Figure S6.** Kaplan-Meier survival analysis of **grades III and IV in training and validation data sets.**

(A-B) The patients in grades III and IV in the training data set.

(C-D) The patients in grades III and IV in the validation data sets. Each group was classified by the 89-gene signature into high and low risk groups. The *p* values were computed by the log-rank test.

**Supplementary Figure S7.** Kaplan-Meier survival analysis of **adjuvant chemotherapy and radiation therapy in the training and validation data sets.**

(A-B) Patients with radiotherapy in the training and validation data sets.

(C-D) Patients with chemotherapy in the training and validation data sets.

(E-F) Patients with combined therapies in the training and validation data sets. Each group was stratified according to chemotherapy, radiotherapy, and combined therapies. The *p* values were computed by the log-rank test.

**Supplementary Figure S8.** Protein interaction network analysis in the 89-gene signature.

Interaction map was generated using the STRING database.

**Supplementary Figure S9. NF-KB, AP-1, and STAT3 networks from Ingenuity pathway analysis.**

Gene networks from IPA showed upstream genes significantly associated with NF-KB, AP-1, and STAT3 pathways.
